# Influence of forward head posture on muscle activation pattern of the trapezius pars descendens muscle in young adults

**DOI:** 10.1038/s41598-022-24095-8

**Published:** 2022-11-14

**Authors:** Yuichi Nishikawa, Kohei Watanabe, Takanori Chihara, Jiro Sakamoto, Toshihiko Komatsuzaki, Kenji Kawano, Akira Kobayashi, Kazumi Inoue, Noriaki Maeda, Shinobu Tanaka, Allison Hyngstrom

**Affiliations:** 1 Faculty of Frontier Engineering, Institute of Science & Engineering, Kanazawa University, Kanazawa, Kakuma-Machi, Kanazawa, Ishikawa 920-1192 Japan; 2 Laboratory of Neuromuscular Biomechanics, School of Health and Sport Sciences, Chukyo University, Nagoya, Japan; 3 Faculty of Advanced Manufacturing Technology Institute, Kanazawa University, Kanazawa, Kanazawa, Ishikawa Japan; 4 Division of Seat Evaluation & Engineering, Toyota Boshoku, Toyota, Aichi Japan; 5 Division of Sports Rehabilitation, Graduate School of Biomechanical and Health Sciences, Hiroshima University, Hiroshima, Hiroshima Japan; 6 Department of Physical Therapy, Marquette University, Milwaukee, WI USA

**Keywords:** Health care, Engineering

## Abstract

Forward head posture (FHP) is a serious problem causing head and neck disability, but the characteristics of muscle activity during long-term postural maintenance are unclear. This study aimed to investigate a comparison of electromyography (EMG) activation properties and subjective fatigue between young adults with and without habitual FHP. In this study, we examined the changes in the spatial and temporal distribution patterns of muscle activity using high-density surface EMG (HD-SEMG) in addition to mean frequency, a conventional measure of muscle fatigue. Nineteen male participants were included in the study (FHP group (n = 9; age = 22.3 ± 1.5 years) and normal group (n = 10; age = 22.5 ± 1.4 years)). Participants held three head positions (e.g., forward, backward, and neutral positions) for a total of 30 min each, and the EMG activity of the trapezius pars descendens muscle during posture maintenance was measured by HD-SEMG. The root mean square (RMS), the modified entropy, and the correlation coefficient were calculated. Additionally, the visual analogue scale (VAS) was evaluated to assess subjective fatigue. The RMS, VAS, modified entropy, and correlation coefficients were significantly higher in the FHP group than in the normal group (*p* < 0.001). With increasing postural maintenance time, the modified entropy and correlation coefficient values significantly decreased, and the mean frequency and VAS values significantly increased (*p* < 0.001). Furthermore, the forward position had significantly higher RMS, correlation coefficient, modified entropy, and VAS values than in the neutral position (*p* < 0.001). The HD-SEMG potential distribution patterns in the FHP group showed less heterogeneity and greater muscle activity in the entire muscle and subjective fatigue than those in the normal group. Excess muscle activity even in the neutral/comfortable position in the FHP group could potentially be a mechanism of neuromuscular conditions in this population.

## Introduction

Forward head posture (FHP) is a head and neck flexion posture that is associated with cervical neck disease and due to several environmental/behavioral factors, it is seen increasingly in young adults. In recent years, the frequency of working from home and attending meetings online has increased rapidly with the spread of novel coronavirus disease^1^, and it has been observed that people are spending more time in a seated position^2^. Prolonged sitting has been suggested as a risk factor for neck pain^3^, and a previous study reported that there is an association between sitting time in total per day and the intensity of neck pain^4^. Furthermore, there has been a potentially harmful increase in the use of smartphones for texting, especially among young people, combined with the increasing prevalence of neck pain^3,5^. The prolonged use of smartphones and personal computers could cause musculoskeletal problems. In a previous study, it was reported that screen viewing time is associated with an increased posture of flexion of the neck and head in children, especially in a sitting position^6^. Knowledge in this area is clinically relevant, as the long-term effects of FHP during adolescence have been suggested to predispose adults to headaches and neck pain^7,8^, and the identification of abnormalities in subjective fatigue and muscle activity in asymptomatic FHP is important in the prevention of head and neck orthopedic problems.

FHP can be associated with key abnormalities in neuromuscular function, such as a lower endurance of the deep neck extensors and flexors, as well as a higher activity of the superficial muscles in adults with neck pain^9^. However, several studies that examine FHP using surface electromyography (EMG) have focused on the standing position in which the head and neck do not touch a pillow or head rest of a seat in people with neck symptoms^10,11^, and no reports have been made in a sitting position, especially the resting position (head leaning against a pillow or other object) in people with asymptomatic FHP. It is important to study this position because it is necessary to maintain the same posture for long periods of time in the resting posture while working at home or while using an economy class seat in airplane travel. Furthermore, several previous studies have examined the assessment of neuromuscular function using surface EMG during several postures in people with FHP. However, a pair of small electrodes is generally used to record surface EMG signals from a muscle of interest, and the detected surface EMG signals can only provide information about a very small portion of the muscle. As a method to estimate motor unit activation or to provide more detailed physiological data, a high-density surface EMG (HD-SEMG) technique has been developed recently that records surface EMG signals from large areas of muscle using multiple two-dimensionally oriented electrodes^12^. Previous studies reported region-specific muscle activity and muscle fatigue in the upper trapezius muscle^13,14^. Consequently, HD-SEMG could be useful to understand neuromuscular function and/or fatigue in people with FHP. However, there are no reports of HD-SEMG applications to head and neck extensor muscles, and the neuromuscular function and/or fatigue properties of FHP remain unclear.

Here, we compared EMG properties and subjective fatigue between young adults with and without FHP. Due to weakness in the deep neck extensors, we hypothesized that the FHP group would show greater subjective fatigue and activity in the muscle that maintains the neck position, i.e., trapezius pars descendens muscle, in a head-leaning sitting posture than the normal group. The results of this study identify early muscle abnormalities in people with asymptomatic FHP and provide some mechanistic insight with regard to FHP-related neck pain, and provide insight into some of the factors contributing to head and neck disorders in FHP. To help us interpret our results, measurements of EMG distribution patterns were performed using HD-SEMG. Other HD-SEMG measures, such as entropy, will be used to provide mechanistic insight.

## Materials and methods

### Participants

**Figure 1:**
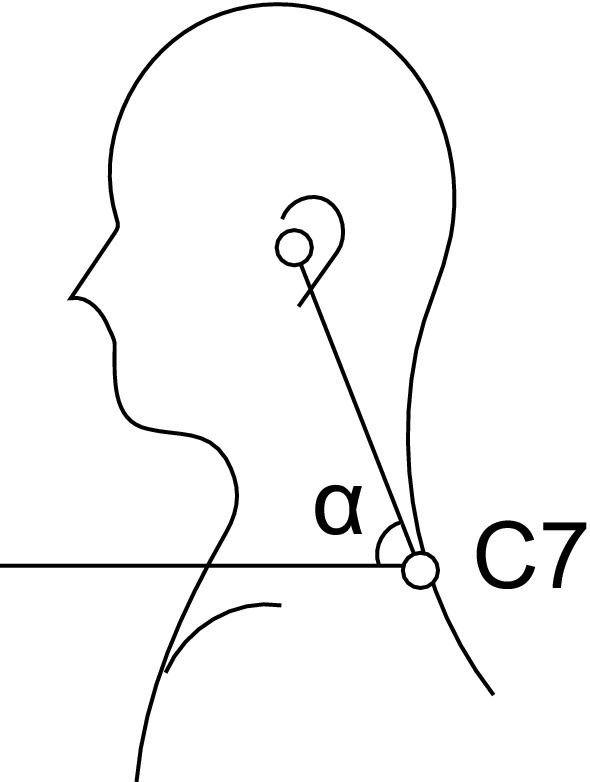
Measurement of the craniovertebral angle. In upright standing posture, the craniovertebral angle (α) was calculated as the angle between the horizontal line passing through C7 and a line extending from the tragus of the ear to C7.

Nineteen young adults were enrolled in this study after signing an informed consent form. All experimental protocols of this study were approved by the Ethics Committee of the Institute of Science and Technology, Kanazawa University (No. 2021-8), and all methods were carried out in accordance with the requirements of the Declaration of Helsinki. The inclusion criteria were age ≥ 20 years old and no neck or shoulder pain. The exclusion criteria were a history of neck and back injury and neurological diseases (Parkinson’s syndrome, dementia, myositis, spinal muscular atrophy, and dystonia). The craniovertebral angle was calculated as the angle between the horizontal line passing through C7 and a line extending from the tragus of the ear to C7 for all participants (Fig. 1)^15^. The FHP group included those with a craniovertebral angle < 53°, n = 9 (age, 22.3 ± 1.5 years; height, 171.6 ± 3.6 cm; weight, 60.6 ± 5.2 kg) and the normal group had a craniovertebral angle > 53°, n = 10 (age, 22.5 ± 1.4 years; height, 173.3 ± 3.6 cm; weight, 63.6 ± 6.1 kg). Determination of 53° as a reference angle was conducted by study Lee et al.^16^, Yib et al.^17^, and Salahzadeh et al. who reported 55° as a normal range and subjects with FHP had a smaller angle than normal subjects.

### Experimental protocols

**Figure 2:**
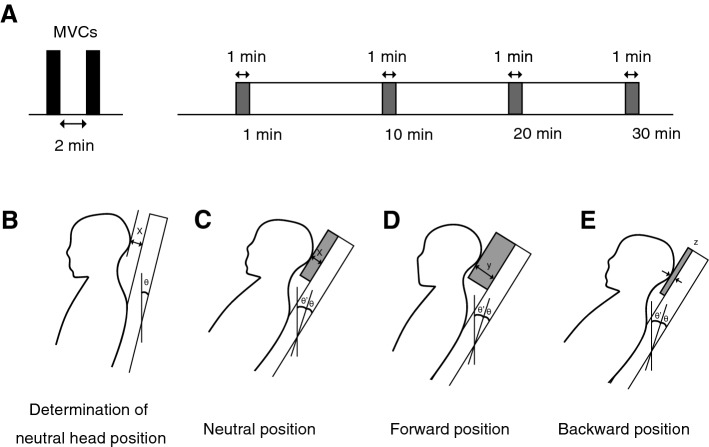
Study protocol and determination of three head positions. (**A**) All participants were asked for the maximal voluntary contraction (MVC) of head extension twice. After MVC measurements, participants held the sitting posture for 30 min, and electromyography measurements were taken for 1 min at the beginning of the posture and for 1 min every 10 min thereafter (gray bar). (**B**) Looking straight ahead and adjusting the seat recline, the most comfortable head and neck position is the neutral head position. X indicates the distance between the back of the head and the seat. (**C**) The neutral position was defined as the posture with the 5° recline folded down from the neutral head position (θ′ = 5°). A pillow with a height of x was fabricated for each participant, and the participant was made to lean against the pillow. (**D,E**) The position with the pillow height 3 cm higher or lower than the neutral position was defined as the + 3 position or − 3 position (y = x + 3 cm and z = x − 3 cm).

All subjects were measured for MVC in the neutral position (see below for details) and then held in the sitting posture for 30 min in three different head postures (neutral, forward, and backward) to examine the influence of head position on muscle activity and fatigue (Fig. 2). The sessions were conducted once each.

In sitting, participants adjusted the seat recline while looking straight ahead and identified the most comfortable head and neck position as the “neutral head position” (Fig. 2B). Next, the seat was reclined 5° (θ′ = 5°), a pillow corresponding to the height of x was prepared, and the participant was instructed to place the back of the head on the pillow and lean back in a neutral position (Fig. 2C). Then + 3 cm (“Forward”) and − 3 cm (“Backward”) from the neutral position were defined (Fig. 2D,E). EMG data were then measured in each of the three head holding positions. The reclining angle (θ′ = 5°) was set to prevent the head from falling forward when in a forward displaced position (+ 3 cm). EMG measurements were taken during the isometric maximal voluntary isometric contraction (MVC) measurement and the first minute of posture maintenance and every 10 min thereafter for 1 min. The EMG data during these periods were used for analysis. All participants held each position (e.g., forward, backward, and neutral) a total of 30 min. The arms were allowed to droop along the trunk, and the knees were placed in a comfortable 70°–90° flexed position. The order of positions was randomized, and the interval between tasks was at least one day to minimize the effects of fatigue.

We adjusted the resistance pad on the cervical device movement arm so that it was at a level that was just superior to the external occipital protuberance. The resistance pad was locked in place, and participants were instructed to perform a series of two MVC attempts against the fixed resistance pad. For the MVC measurement, the participant was instructed to perform head extension as hard as possible for 5 s without force in the hip and shoulders. To prevent the subject from exerting force in the hip and shoulders, the subject was asked to sit deeply in the seat so that the hip would not lift, and the arms were kept relaxed. Each of these efforts was held for a two-minute rest period between each of these two efforts (Fig. 2A). For each participant, we treated the highest EMG voltage (MVC-Max) observed in these two MVC trials as the maximum voltage that the participant could attain during an MVC effort. Additionally, we measured the visual analogue scale (VAS) at each posture at 30 min as a subjective fatigue assessment. Subjective fatigue was assessed for fatigue related to the head and neck area. The VAS was measured on a scale of 0 to 100, with 0 defined as not fatigued and 100 defined as maximally fatigued^18^.

### EMG recording

**Figure 3:**
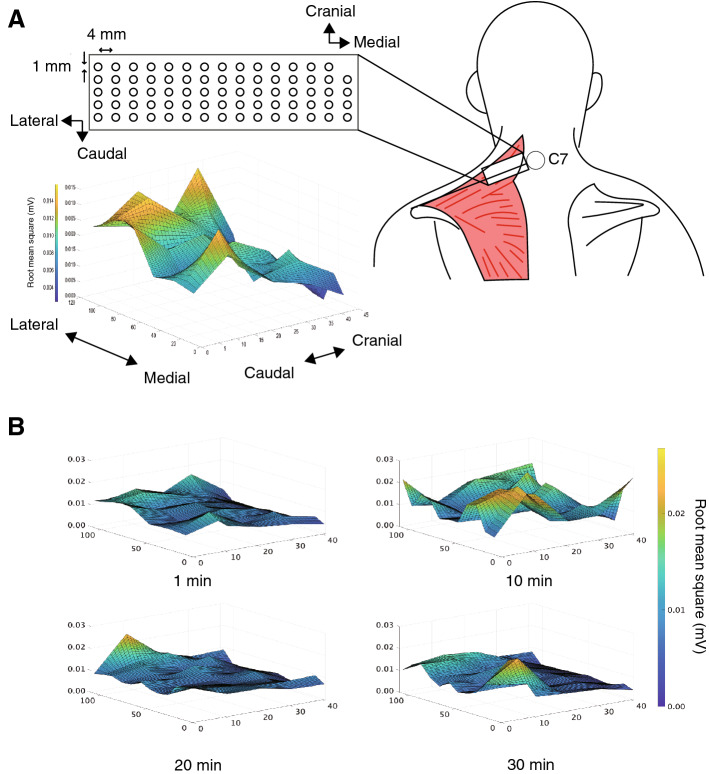
Electrode placement and color map of the representative high-density surface electromyography (HD-SEMG) in each period during posture maintenance. (**A**) The 64-electrode array was placed on the trapezius pars descendens muscle (electrode diameter; 1 mm and interelectrode distance; 4 mm). Topographic map of the root mean square value of the bipolar EMG recorded at the neutral position during posture maintenance. (**B**) Illustration of a color map of the representative HD-SEMG in a neutral position for each period during posture maintenance in a young adult (age 21 years).

The 64-electrode grid (1 mm, diameter; 4 mm, intra-electrode distance, GR04MM1305, OT Bioelettronica, Turin, Italy) was placed on the trapezius pars descendens muscle of the dominant side (Fig. 3A). The medial side of the electrode grid was placed at the lateral side of C7 and affixed on a line connecting C7 and the acromion. After cleaning the skin (80% alcohol), an electrode grid was attached to the muscle surface with a two-adhesive sheet (KIT04MM1305, OT Bioelettronica) with a conductive paste (Elefix ZV-181E, NIHON KOHDEN, Tokyo, Japan)^19^. The seventh cervical spine was placed with a ground electrode. Monopolar HD-SEMG signals were recorded using a 16-bit AD converter (Quattrocento, OT Bioelettronica, sampling frequency at 2048 Hz), amplified at a 150 gain and filtered at a 10–500 Hz off-line bandpass^20,21^. MATLAB software (MATLAB 2021b, Math Works GK, MA, USA) was used to analyze EMG signals.

### Data processing

A total of 59 bipolar EMG signals were calculated from adjacent electrodes (12 bipolar recordings in each row except the upper row, which had 11 electrode pairs, Fig. 3A). The root mean square (RMS) and mean frequency were calculated for each electrode and the mean values were calculated for all electrodes from all of the data at each period (1 min, 10 min, 20 min, and 30 min). Furthermore, the RMS of the MVC was calculated from 1 s of data centered on the maximum voltage during MVC. The RMS value was normalized to the MVC value. RMS and mean frequency were computed as follows:

$$RMS=\sqrt{\frac{1}{N}{\sum }_{i=1}^{N}{({EMG}_{i})}^{2}},$$

where *N* is the length of the signal, *EMG* is the EMG signal, and *i* is the *i*th sample.

$$Mean \, frequency=\int_{0}^{\infty }f\cdot P\left(f\right)df/\int_{0}^{\infty }P\left(f\right)df,$$

where *f* is the sampling frequency and *P* is the power at that frequency.

To characterize the heterogeneity in the spatial distribution of the HD-SEMG potential at each period, we determined the modified entropy and correlation coefficient. The modified entropy was calculated for 59 RMS values at MVC and each period, as performed in a previous study^14^.

$$E= -{\sum }_{i=1}^{59}p{\left(i\right)}^{2}{\text{log}}_{2}{p\left(i\right)}^{2},$$

where *p(i)*^2^ is the RMS value square of electrode *i*, which is normalized by the total of 59 RMS values over a given period. The correlation coefficient was calculated using 59 RMS pairs of the same region for 1 min compared to 10 min, 20 min, and 30 min (Fig. 3B).

The reduced modified entropy indicates an increase in the heterogeneity of the spatial distribution of the HD-SEMG potential in the electrode grid. A reduction in the correlation coefficients indicates an increase in the temporal distribution of the HD-SEMG potential. Changes in the spatial and temporal distribution patterns of the HD-SEMG potential show relative adaptations to muscle activity intensity during contraction and may be attributed to changes in the peripheral characteristics of the muscle or to the control of the motor unit within the muscle^22^.

### Statistical analysis

All statistical analyses were conducted using Stata ver. 17 (Stata Corp LLC, Texas, USA), and GraphPad Prism version 8 (GraphPad Software Inc, California, USA) was used to generate graphics. Shapiro–Wilk tests were conducted on all data to ensure normality. Separate unpaired *t-*tests were used to detect differences in age, height, and weight between the FHP and normal groups. The generalized linear mixed-effects model with random intercepts and random slopes with Bonferroni’s multiple comparison test as a post hoc test was applied to analyze the normalized RMS, modified entropy, correlation coefficients, VAS, and mean frequency. The explanatory variables were group (FHP and normal), period (1 min, 10 min, 20 min, and 30 min), and position (neutral, forward, and backward). The significance level was *p* < 0.05.

## Results

Age, height, and weight were not different between the groups (*p* = 0.8021, *p* = 0.3064, and *p* = 0.2566, respectively).

There was a significant interaction effect of group $\times$ period $\times$ position for VAS (*F* = 2.80, *p* = 0.0100, *η*^2^ = 0.141), modified entropy (*F* = 5.84, *p* < 0.0001, *η*^2^ = 0.256), and the correlation coefficient (*F* = 2.25, *p* = 0.0359, *η*^2^ = 0.117); on the other hand, the normalized RMS (*F* = 0.15, *p* = 0.9891, *η*^2^ = 0.008) and mean frequency (*F* = 0.235, *p* = 0.9650, *η*^2^ = 0.013) did not show a significant interaction effect of group $\times$ period $\times$ position.

**Figure 4:**
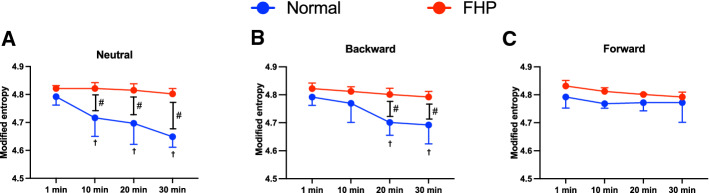
Comparison of modified entropy between groups in neutral (**A**), backward (**B**), and forward (**C**) postures. The forward head posture (FHP) group showed significantly higher values at 10 min, 20 min, and 30 min in the neutral posture, and significantly higher values at 20 min and 30 min in the backward posture. Furthermore, the normal group showed a significant decrease over time during posture maintenance in the neutral and backward postures. Data showed median ± 95% CI. ^#^*p* < 0.05, FHP vs. normal; ^†^*p* < 0.05, compared with 1 min.

**Figure 5:**
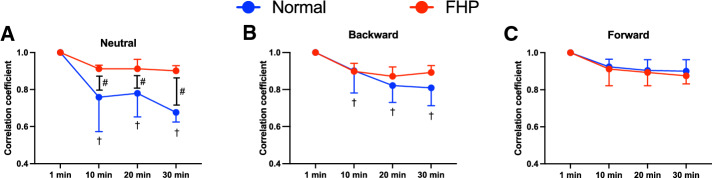
Comparison of correlation coefficients between groups in neutral (**A**), backward (**B**), and forward (**C**) postures. In the neutral posture, the forward head posture (FHP) group showed a significantly higher correlation coefficient than the normal group (**A**). The normal group showed a significant decrease over time during posture maintenance in the neutral and backward postures (**A,B**). In the forward posture, each group did not show a significant difference between each period (**C**). Data showed median ± 95% CI. ^#^*p* < 0.05, FHP vs. normal; ^†^*p* < 0.05, compared with 1 min.

**Figure 6:**
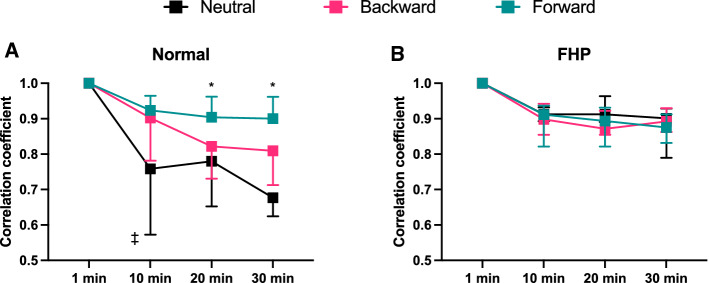
Comparison of modified entropy between each posture in normal (**A**) and forward head posture (FHP) (**B**) groups. In the forward posture, the normal group showed significantly higher levels than in the neutral and backward postures (**A**). On the other hand, the FHP group did not show a significant difference between each posture. Data showed median ± 95% CI. *p < 0.05, compared with neutral; ^†^*p* < 0.05, compared with backward.

**Figure 7:**
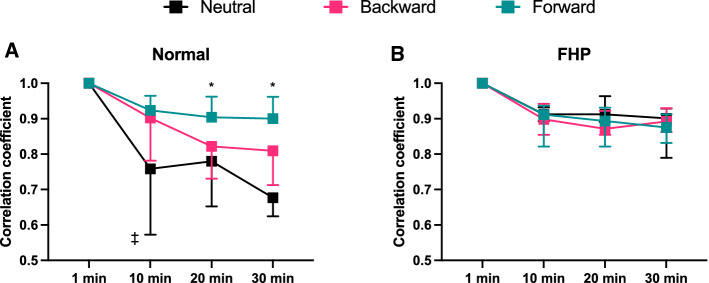
Comparison of correlation coefficients between each posture in the normal (**A**) and forward head posture (FHP) (**B**) groups. The normal group showed significantly higher correlation coefficients at 20 min and 30 min in the forward posture than in the neutral posture (**A**). Furthermore, the neutral posture showed a significantly lower correlation coefficient at 10 min than the backward and forward postures in the normal group. On the other hand, the FHP group did not show a significant difference between each posture (**B**). Data showed median ± 95% CI. *p < 0.05, compared with neutral; ^‡^*p* < 0.05, compared with backward and forward.

The modified entropy and correlation coefficient values were significantly lower at 10 min, 20 min, and 30 min in the normal group in the neutral position than in the FHP group (*p* < 0.001) (Figs. 4A, 5A). Furthermore, the normal group showed significantly lower modified entropy at 20 min and 30 min in the backward position than the FHP group (*p* < 0.0001) (Fig. 4B). On the other hand, the forward position did not show a significant difference at each period between the groups (Figs. 4C, 5C). The normal group showed significantly decreased modified entropy and correlation coefficients over time in neutral and backward positions compared with 1 min (*p* < 0.01) (Figs. 4A,B, 5A,B), but the forward position did not show a significant difference between each period (Figs. 4C, 5C). The normal group showed significantly higher modified entropy at 10 min, 20 min, and 30 min in the forward position than in the neutral position (*p* < 0.0001), and significantly higher modified entropy at 20 min (*p* = 0.001) and 30 min (*p* < 0.0001) in the forward position than in the backward position (Fig. 6A). The correlation coefficients in the normal group were significantly higher at 20 min (*p* = 0.005) and 30 min (*p* < 0.0001) in the forward position than in the neutral position. Furthermore, the normal group showed a significantly lower correlation coefficient at 10 min in the neutral position than in the backward (*p* = 0.049) and forward (*p* < 0.0001) positions (Fig. 7A). On the other hand, the FHP group did not show significant differences in modified entropy and correlation coefficients among postures (Figs. 6B, 7B).

**Figure 8:**
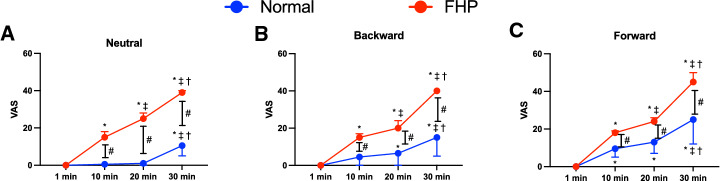
Comparison of visual analogue scale (VAS) scores between groups in neutral (**A**), backward (**B**), and forward (**C**) postures. The forward head posture (FHP) group showed significantly higher VAS scores at each period in all postures than in the normal group. The FHP and normal groups showed significantly increased VAS scores over time during posture maintenance in each posture. Data showed median ± 95% CI. ^#^*p* < 0.05, FHP vs. normal; *p < 0.05, compared with 1 min; ^‡^*p* < 0.05, compared with 10 min; ^†^*p* < 0.05, compared with 20 min.

**Figure 9:**
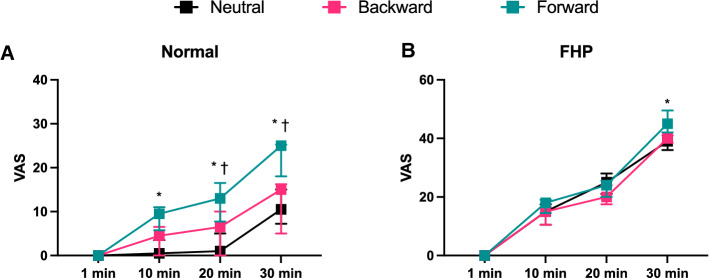
Comparison of visual analogue scale (VAS) scores between each posture in the normal (**A**) and forward head posture (**B**) groups. The normal group showed significantly higher VAS scores in the forward posture than in the neutral and backward postures during posture maintenance (**A**). On the other hand, the forward head posture (FHP) group showed significantly higher VAS scores in the forward posture only at 30 min in the forward posture than in the neutral posture (**B**). Data showed median ± 95% CI. *p < 0.05, compared with neutral; ^†^*p* < 0.05, compared with backward.

The VAS score was significantly lower at 10 min, 20 min, and 30 min in the normal group at each position than in the FHP group (*p* < 0.0001) (Fig. 8). The FHP group showed a significantly increased VAS score over time compared with 1 min in each posture (*p* < 0.0001,) (Fig. 8). The normal group showed a significantly increased VAS score over time compared with 1 min in the forward posture (*p* < 0.0001) (Fig. 8C). The normal group showed significantly higher VAS scores at 10 min (*p* = 0.001), 20 min (*p* < 0.0001), and 30 min (*p* < 0.0001) in the forward position than in the neutral position and significantly higher VAS scores at 20 min (*p* = 0.004) and 30 min (*p* < 0.0001) in the forward position than in the backward position (Fig. 9A). The FHP group showed a significantly higher VAS score at 30 min in the forward position than in the neutral position (*p* = 0.002) (Fig. 9B).

**Figure 10:**
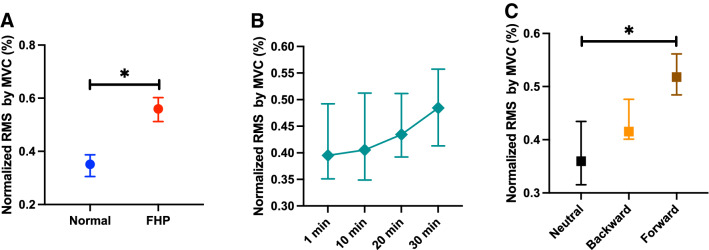
Comparison of normalized root mean square (RMS) values between groups (**A**), period (**B**), and each posture (**C**). The forward head posture (FHP) group showed a significantly higher normalized RMS values by maximal voluntary contraction (MVC) than the normal group (**A**), but the normalized RMS values did not show a significant change (**B**). The neutral posture showed a significantly lower normalized RMS value than the forward posture (**C**). Data showed median ± 95% CI. *p < 0.05.

The FHP group showed a significantly higher normalized RMS value than the normal group (*p* < 0.0001, *η*^2^ = 0.141) (Fig. 10A). The normalized RMS value did not show a significant difference among the periods (1 min vs. 10 min; *p* = 1.000, 1 min vs. 20 min; *p* = 1.000, 1 min vs. 30 min; *p* = 0.559, 10 min vs. 20 min; *p* = 1.000, 10 min vs. 30 min; *p* = 1.000, 20 min vs. 30 min; *p* = 1.000) (Fig. 10B). The neutral position showed a significantly lower normalized RMS value than the forward head position (*p* = 0.006), but there was no significant difference between neutral and backward positions (*p* = 0.307) or backward and forward (*p* = 0.401) (Fig. 10C).

**Figure 11:**
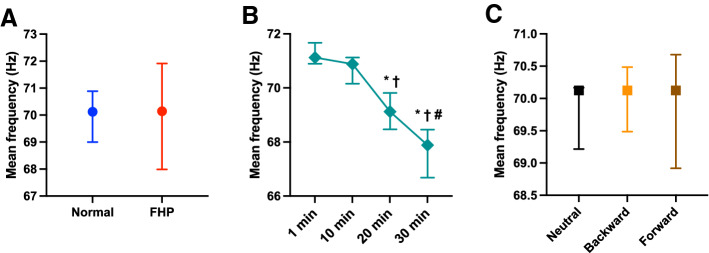
Comparison of the mean frequency between groups (**A**), period (**B**), and each posture (**C**). There was no significant difference in the mean frequency between the forward head posture (FHP) and normal groups (**A**). The mean frequency showed a significant decrease over time (**B**). There was no significant difference in the mean frequency of each posture (**C**). Data showed median ± 95% CI. *p < 0.05, compared with 1 min; ^†^*p* < 0.05, compared with 10 min; ^#^*p* < 0.005, compared with 20 min.

In the mean frequency, there was no significant difference between the normal and FHP groups (*p* = 0.5633, *η*^2^ = 0.03) (Fig. 11A). The mean frequency was significantly higher at 1 min than at 20 min and 30 min (*p* < 0.0001), that at 10 min was significantly higher than that at 20 min and 30 min (*p* < 0.0001), and that at 20 min was significantly higher than that at 30 min (*p* < 0.0001) (Fig. 11B). On the other hand, there was no significant difference between each position (neutral vs. backward; *p* = 1.000, neutral vs. forward; *p* = 0.432, backward vs. forward; *p* = 0.415) (Fig. 11C).

## Discussion

This study compared the spatial and temporal distribution patterns of HD-SEMG in the trapezius pars descendens muscle between FHP and normal conditions. The primary novel results were as follows: the FHP group exhibited a (1) greater RMS amplitude, (2) lower heterogeneity, (3) smaller temporal changes, and (4) greater quantitative/subjective fatigue (e.g., mean frequency and VAS score) during the long-term sitting position than the normal group. Of particular importance, we found greater muscle activity in the FHP group even in the neutral position. Furthermore, the mean frequency analysis used in this study was able to detect muscle fatigue, while the spatial and temporal distribution analysis of muscle activation was able to identify abnormalities in muscle activity between different postures in each group. These findings suggest that, in addition to frequency analysis, analysis of the distribution of muscle activation patterns can be used to identify more detailed fatigue in the head and neck region.

In this study, we measured muscle activity during three different positions (e.g., neutral, forward, and backward) for both the FHP and normal groups and compared changes in the temporal and spatial distribution patterns of trapezius pars descendens muscle activity and quantitative/subjective fatigue. Previous studies have shown that head displacement forward or backward from a neutral position muscle’s EMG activity increased in trapezius pars descendens muscle^23,24^. These previous findings are consistent with the results of this study showing that compared to the neutral position, the forward position exhibited a higher RMS value in the normal group. Interestingly, the FHP group showed significantly higher normalized RMS values than the normal group. Previous studies by Hollgren et al. demonstrated that voluntary head retraction and/or protrusion results in a statistically significant increase in EMG activity in the posterior rectus capitis muscle, resulting in eccentric and/or concentric contractions^23,24^. The deep muscle of the higher spine functions to stabilize the head and neck, maintain posture, and protect against movements caused by unexpected external forces^25^. These findings suggest that deviation from the neutral position of the head and neck is associated with increased muscle activity. Importantly, our results showed that muscle activity increased despite leaning the head and neck against a headrest, and for the FHP group was found to have highly subjective fatigue even in comfortable head and neck positions. These findings suggest that deviation from the neutral position could potentially induce headache and neck disorders if the same posture is held for long periods of time, and with respect to FHP, prolonged postural holding, including the neutral position, can cause headache and neck health problems.

In neutral and backward postures, the modified entropy and correlation coefficient were significantly lower in the normal group than in the FHP group. Furthermore, these variables were significantly decreased over time during posture maintenance in the normal group. The mean frequency was found to decrease with increasing postural retention time, but there were no differences between groups and postures. The modified entropy and correlation coefficient assess the temporal and spatial distribution of muscle activity, and changes in HD-SEMG potential distribution patterns indicate relative adaptations in the intensity of activity within muscle regions during contraction and may be attributed to variations in peripheral properties or in the control of motor units within a muscle^26,27^. A previous study reported a relationship between the spatial distribution of muscle activity and endurance time, with a greater spatial distribution of muscle activity being associated with less fatigue^14^. Consistent with this previous finding, our results showed significantly lower subjective fatigue in the normal group among head positions than in the FHP group. These results indicate that the FHP group has problems with the adaptive control function of muscle activity in postural retention within a muscle. Previously, it was investigated whether there is a relationship between head posture and neck pain and whether FHP differs between neck pain and asymptomatic people. There was a significant difference between asymptomatic and symptomatic adults with FHP, as determined by the results^28^. Furthermore, increased FHP can be associated with lower endurance of the deep neck extensors and flexors as well as greater activity of the superficial muscles in adults with neck pain^9,29^. These findings support the results of this study that people with FHP exhibit greater subjective fatigue and muscle activity. Importantly, this study found significant differences in subjective fatigue and muscle activity, although only young adults without head and neck pain were included in this study. The long-term effects of reduced flexibility and endurance of neck muscles during adolescence have been suggested to predispose adults to headache and neck pain^7^, and our results of the spatial and temporal distribution patterns of muscle activity analyses used in this study indicate early detection of abnormal muscle activity caused by postural displacement of the head and neck. Our results also showed that the FHP group was not effective in reducing head and neck fatigue when changing head position. Therefore, people with FHP may need to reduce fatigue in ways other than adjusting the position of the head and neck position (e.g., using armrests or changing the shape of the pillow). In the future, when providing therapeutic intervention for people with FHP, it may be necessary to clarify the comfortable position for people with FHP.

This study has several limitations. First, this study included only young males. Potential confounders that can influence neck pain and FHP, such as age and sex, must be controlled, and female participants and a wider age range should be included to clarify FHP and abnormal muscle activity. Second, this study measured only the trapezius pars descendens muscle. The muscle activity control mechanisms of not only the extensor muscles of the head and neck but also the flexor muscles play an important role in maintaining the posture of the head and neck. In the future, including the head and neck flexor muscles in the measurement will lead to a greater understanding of functional abnormalities in FHP.

In conclusion, we compared the spatial muscle distribution and quantitative/subjective fatigue during postural retention between the FHP and normal groups. Compared with the normal group, the FHP group exhibited greater subjective fatigue and muscle activity and lower spatial and temporal changes in muscle activation patterns. These findings suggest that long-term neck displacement may be a potential factor contributing to neck pain. This study revealed that head and neck position had little effect on muscle activity and fatigue in the FHP group, suggesting that other interventions, such as the use of arm rests or adjusting the buttock position, are important for FHP. In the future, it is necessary to examine methods to reduce head and neck muscle strain in the FHP group to prevent head and neck disorders.

## Data Availability

The datasets analyzed in this study are available from the corresponding author on reasonable request after approval by institutional authorities.
